# Development of a Bioactive Dental Barrier Membrane Based on PCL/Collagen and PVA/Hydroxyapatite Layers with Amoxicillin-Loaded Electrosprayed Coating

**DOI:** 10.3390/pharmaceutics18050610

**Published:** 2026-05-17

**Authors:** Hilal Gülsena Nur Akkus, Ayse Betül Bingol, Büsra Oktay, Buse Ozsan, Ahmet Akif Kızılkurtlu, Azime Erarslan, Fatih Ciftci, Cem Bülent Ustündag

**Affiliations:** 1 Faculty of Chemistry-Metallurgy, Department of Bioengineering, Yildiz Technical University, Istanbul 34220, Turkey; 2 Faculty of Arts and Sciences, Department of Chemistry, Yildiz Technical University, Istanbul 34220, Turkey; 3 Faculty of Engineering and Natural Sciences, Department of Biomedical Engineering, Atlas University, Istanbul 34403, Turkey; 4 Faculty of Engineering, Department of Biomedical Engineering, Fatih Sultan Mehmet Vakıf University, Zeytinburnu, Istanbul 34015, Turkey; fciftci@fsm.edu.tr; 5 BioriginAI Research Group, Department of Biomedical Engineering, Fatih Sultan Mehmet Vakıf University, Zeytinburnu, Istanbul 34015, Turkey; 6 Biomedical Electronic Design Application and Research Center (BETAM), Fatih Sultan Mehmet Vakıf University, Zeytinburnu, Istanbul 34015, Turkey; 7 Health Biotechnology Joint Research and Application Center of Excellence, Yildiz Technical University, Istanbul 34220, Turkey

**Keywords:** biomaterials, dental membrane, electrospinning, electrospray, drug, polymer

## Abstract

**Background/Objectives**: Guided bone regeneration (GBR) in dental applications requires scaffolds that possess balanced mechanical strength, controlled biodegradability, and excellent biological performance; therefore, this study aims to develop and evaluate a multilayered biofunctional dental membrane designed to enhance mechanical, biological, and antibacterial performance. **Methods**: The multilayered membrane was fabricated using sequential electrospinning and electrospraying techniques to form a polycaprolactone (PCL)/Collagen first layer and a polyvinyl alcohol (PVA)/Collagen/Hydroxyapatite (HAp) second layer, topped with a final electrospray coating of PVA/Amoxicillin. Characterization was performed via SEM, FTIR, and EDS, followed by evaluations of tensile properties, swelling behavior, hydrolytic degradation, in vitro drug release, disk diffusion antibacterial activity against *Staphylococcus aureus* and *Escherichia coli*, and 7-day L929 fibroblast cytocompatibility (ANOVA/Tukey, *p* < 0.05). **Results**: SEM, FTIR, and EDS analyses confirmed uniform nanofiber morphology, homogeneous HAp distribution, and successful integration of bioactive compounds. The membrane exhibited a maximum tensile strength of 15.17 N, strain of 25.24%, and stress of 2.16 MPa, while swelling reached ~100% within 2 h and degradation stabilized around 4% weight loss after 48 h. Drug release profiles showed a rapid amoxicillin release in the first 50 min, plateauing at approximately 4.5 mg/L by 350 min, with distinct antibacterial inhibition zones, and the PCL/Col–PVA/Col/HAp–PVA/Amox group demonstrated the highest cell viability (~140%) after 7 days, significantly exceeding the control groups (*p* < 0.01). **Conclusions**: These quantitative findings validate the fabricated multilayered membrane’s potential as a mechanically robust, biodegradable, antibacterial, and bioactive scaffold for advanced guided bone regeneration in dental applications.

## 1. Introduction

Dental membranes are used in dental surgery applications, in predictable bone grafting applications, filling bone defects, and dental implant placement [1,2,3]. Depending on the application, resorbable and non-resorbable membranes can be used. Considering the shortcomings of other treatment methods, the dental membrane produced in this study has a double-layered structure that provides support for the defects after filling, supports bone healing, and prevents bacterial growth [4,5]. FDA-approved, resorbable, and biocompatible polymers were used to create the scaffold structure [6,7]. The emergence of tissue engineering sheds light on the treatment of new patients in a multidisciplinary field. It includes dentistry, tissue engineering for the regeneration of non-dental tissues and dental tissues, as well as supporting structures [7,8]. In dentistry, tissue engineering is applied in three main areas for the regeneration of teeth. These are pulp-dentin complex, regeneration, bone regeneration, and periodontal tissue regeneration. While performing regenerative applications for dental diseases, various studies are carried out to repair and regenerate the tooth structure by utilizing the fields of engineering and biology [9,10,11].

The dental membrane consists of a double-layered structure with a nanofiber structure produced using the electrospinning method. The first layer contains polyvinyl alcohol (PVA) [12], hydroxyapatite (HAp) [13] and collagen (Col) [14,15], and the second layer contains polycaprolactone (PCL) [16] and Col. The double-layered structure will help to control the degradation times of the layers and contribute to a controlled treatment. HAp is present in teeth, dentin and enamel structure, and bones [17,18]. In addition, it is the main component and essential mineral of both bone and teeth [19,20]. Since it has very good biocompatibility, it is a material that can be widely used instead of bone in dental procedures today. In this study, electrospray with PVA and Amoxicillin (Amox) [21,22] the solution was applied on the double layer to provide antibacterial properties for the membrane scaffold produced in this study. In this way, the dental membrane will also prevent the risk of infection during treatment. With this method, which is a topical application, the drug will be used in low doses, and the side effects caused by the drug will be reduced. At this point, it is essential to distinguish between the two techniques used in this hybrid fabrication: while electrospinning utilizes high-viscosity solutions to produce continuous structural nanofibers, electrospraying employs lower polymer concentrations to generate discrete micro-particles. This strategic combination allows the electrospun layers to provide mechanical stability and bioactivity, while the electrosprayed top layer ensures a high surface area for efficient drug delivery. Since absorbable polymers are used in the production of dental membranes, the treatment will be completed without the need for additional surgical procedures after the application. This will positively affect the patient’s quality of life, shorten the duration of treatment, and reduce the patient’s economic burden.

Current dental membranes in the literature primarily focus on single-layer fibrous structures or dense films, which often struggle to balance mechanical rigidity with high drug-loading efficiency. While recent studies have utilized electrospinning for scaffold fabrication, achieving a controlled release of antibiotics without compromising the scaffold’s structural integrity remains a challenge. The novelty of this work lies in the synergistic combination of two distinct techniques: forming a dual-layered, bio-mimetic PCL/Collagen and PVA/Col/HAp nanofibrous base through electrospinning to ensure mechanical robustness (15.17 N tensile force) and subsequently applying an amoxicillin-loaded coating via electrospraying. This hierarchical design not only provides a high surface area for rapid antibacterial action (reaching ~4.5 mg/L amoxicillin) but also optimizes the degradation profile of each layer to match the physiological needs of guided bone regeneration.

After completing the manufacturing of the dental membrane, its structure was analyzed using various techniques, including molecular interaction (FTIR), mechanical (tensile-strength test), morphological (microscope), bacterial analysis, and in vitro cytotoxicity. In the SEM analysis for the double-layer membrane structure, it was observed that the structure has a nanofiber structure, and the fiber diameters are equal and homogeneously distributed. It is also seen that the two membrane layers come together harmoniously, and there is a bonding between the layers. The antibacterial drug-loaded particulate structures formed on the double-layer membrane by the electrospray method were examined by the SEM method, and it was seen that they were produced appropriately. When the mechanical tests are examined, it is seen that the double-layer structure has a strength of approximately 15 Newton force and has a high load-carrying capacity. The average strain value was measured as 25.24%, and this value shows that the membrane is flexible. The analysis for each layer of the membrane structure showed that the structure is mechanically suitable and will be sufficient, and can be used in dental membrane applications. It was observed that the water retention capacity of the membrane structure increased rapidly over time to approximately 100% and then stabilized. In addition, when the drug release profile of the electrosprayed amoxicillin particle double-layer membrane was examined, it was observed that initially the drug was released rapidly in the first 50 min, then it slowed down and reached equilibrium. Also, the zone diameters of the membrane against Gram-positive and Gram-negative bacteria were consistent, and the effect on bacteria was proven. In addition, the cellular biocompatibility and cytotoxicity profiles of the produced membranes, both individually and together with the multilayer structure, were examined in detail. It was observed that PCL/Col membrane and PVA/Col/HAp membranes were also biocompatible separately, while the PCL/Col-PVA/Col/HAp-PVA/Amox. group membrane structure showed the highest proliferation with a cell viability value close to 140%. As a result, it has been proven that the fabricated multilayer biofunctional membrane provides a suitable environment for cellular growth and adhesion. This study aims to provide researchers with a basis for future studies.

## 2. Material Method

### 2.1. Materials

This study came true using the chemicals and devices; PCL (Interlab, Istanbul, Turkey) (Mw 80,000), PVA (Interlab) (Mw 84,000–89,000 g/mol), Collagen (Interlab), Hydroxyapatite, Amoxicillin, Distilled water, Acetic acid, Electrospin, magnetic stirrer (Heidolph-MR3000, Istanbul, Türkiye), lab balance (Weightlab instruments WSA224, Istanbul, Turkey), Thermo Shaker, Optical microscope (Olympus DP27, Olympus, Tokyo, Japan), FT-IR (Tensor 27, BRUKER, Berlin, Germany), UV-Vis spectrophotometer (752N Plus, Shanghai, China), Tensile test machine (SHIMADZU, EZ-LX, Kyoto, Japan), and Micrometer (Mitutoyo Absolute, Aurora, IL, USA).

### 2.2. Preparation of PCL-Collagen Solution and Production of the First Layer by Electro-Spinning Device

In this study, 18% PCL:Acetic acid solution was prepared as the first layer by stirring in a magnetic stirrer for 24 h. Collagen was added into the solution in a ratio of 9:1 PCL:Col and mixed at room temperature in a magnetic stirrer. The mixture was allowed to stir for 2 h until homogeneous. The prepared PCL:Col solution was filled into a syringe. A 21-G needle was attached to the syringe. After placing in the electrospinning device, the device parameters were set to 10 cm distance, 20 kV, and 1 mL/h. For 2 h, the device was operated with the lid closed to produce the membrane. For the production, studies were carried out with 1:1 PCL:Col, 3:1 PCL:Col, and 9:1 PCL:Col ratios, and it was seen that the most suitable ratio for membrane production was 9:1 for PCL:Col due to phase separations in the solution.

Optimization was made with an 18-G needle, and production was continued with a 21-G needle for a thinner fiber size. Electrospinning device parameters were determined with 5, 10, 15 cm distances; (0.5), 1, and 1.5 mL/h speeds; 15, 20, 25 kV voltage, and the most suitable parameters were determined as above so that no bead structure would form and no wet membrane would be made.

### 2.3. Preparation of the PVA-Collagen-Hydroxyapatite Solution and Production of the Second Layer via Electrospinning Device

In this study, the second layer was prepared using a 15% PVA:Distilled water solution, which was mixed with a magnetic stirrer at 70–75 °C for 4 h. Collagen was added to the solution in a 3:1 ratio of PVA to Col. The amount of Hydroxyapatite added was 1% of the volume of the distilled water used. The solution was allowed to mix with the magnetic stirrer at room temperature until it became homogeneous.

The prepared PVA/Col/HAp solution was then poured into a syringe. A 21-G needle was attached to the syringe. After placing it into the electrospinning device, the device parameters were set to a 15 cm distance, 20 kV voltage, and 1.5 mL/h flow rate. Membrane production was carried out for 2 h with the device lid closed (This production was performed on top of the first layer. For characterization purposes, productions were carried out separately). For production, studies were conducted using 12%, 15%, and 20% PVA with a 3:1 PVA:Col ratio, and it was observed that due to the concentration of PVA, the most suitable ratio for membrane production was 15%.

Optimization was initially performed using an 18-G needle; however, to achieve finer fiber sizes, production was continued using a 21-G needle. Electrospinning device parameters were tested at distances of 5, 10, and 15 cm; flow rates of 0.5, 1, and 1.5 mL/h; and voltages of 15, 20, and 25 kV. The most suitable parameters were determined as above, ensuring that bead structures did not form and wet spinning did not occur.

### 2.4. Electrospraying with PVA-Amox

A 4% PVA solution in distilled water was mixed with a magnetic stirrer at 70–75 °C for 4 h. Once the solution reached room temperature, Amox was added at 2% of the PVA content and mixed until fully dissolved.

The prepared PVA-Amox solution was poured into a syringe. A 21-G needle tip was attached to the syringe. After placing it in the electrospinning device, the parameters were set to a 15 cm distance, 20 kV voltage, and 0.5 mL/h flow rate. Electrospraying was carried out for 2 h with the device lid closed. (This production was applied on top of the two previous layers. For characterization purposes, productions were conducted separately.)

Studies were conducted using 8% and 4% PVA, and an electrospray structure was successfully achieved with 4% concentration.

Production trials were performed using electrospinning device parameters at distances of 5, 10, and 15 cm; flow rates of 0.5, 1, and 1.5 mL/h; and voltages of 15, 20, and 25 kV. The most suitable parameters were determined as listed above, ensuring that an electrospray structure formed and wet deposition did not occur.

### 2.5. Chemical Analysis with FTIR (Fourier Transform Infrared Spectroscopy)

FTIR analysis was used to characterize the chemical properties of polymeric nanoparticles. This study identified the chemical bond structure and functional group of the sample formulation using an FT-IR device (Tensor 27, BRUKER, Berlin, Germany) in the 4000–400 cm^−1^ range.

### 2.6. Mechanical Strength Test (Tensile Test)

A tensile testing machine (SHIMADZU, EZ-LX, Japan) was used for mechanical testing. Prior to the tensile test, the thickness of the membranes was measured with a digital micrometer (Mitutoyo MTI Corp., Aurora, IL, USA) at the beginning, middle, and end (height, thickness, diameter), and the average thickness was measured. This value was entered into the device. Each specimen was placed in the relevant compartment of the device, and measurements were performed. For the tensile test, the speed was set to 5 mm/min, and a 0.1 N force [23].

### 2.7. Morphologic Analysis with SEM-EDS-Maps and Optical Microscope

A morphological characterization analysis was performed using a basic optical microscope (Olympus DP27, Japan) for the nanoparticulation with electrospraying. The structure, geometry, and homogeneity of the spherical particles of the produced sample were optimized with optical microscope analysis. Morphological characterization of produced nanoparticles was performed using the SEM (Thermo Scientific Apreo 2 S LoVac, Waltham, MA, USA) device at an accelerating voltage of 10 kV. Samples were cut into suitable sizes, and a very thin (nano-level) gold-palladium (Au-Pd) coating was applied to the samples under high vacuum for observation before imaging. To chemically analyze materials examined with SEM, the device is equipped with an EDS system. SEM and EDS analyses are typically performed simultaneously, allowing point-specific chemical analysis.

In the system, secondary and backscattered electrons are used for morphological imaging, while X-rays are used to identify and quantify elements at detectable concentration levels.

The detection limit for an element in EDS depends on the surface condition of the sample. A smoother surface lowers the detection limit. EDS can detect major and minor elements in concentrations above 10 wt% and 1–10 wt%, respectively. For bulk materials, the detection limit is approximately 0.1 wt%. Therefore, elements present in very low concentrations (e.g., below 0.01 wt%) cannot be detected with EDS.

Optical microscopy can provide a direct image of the structure and, when used with staining techniques, can reveal the alignment of fibers within the material. Reflected and transmitted light microscopy provide information about surface characteristics. In reflected light microscopy, the sample is polished to create a flat surface and placed under the microscope lens for imaging. In transmitted light microscopy, the analysis depends on light passing through the sample, so samples must be optically transparent or semi-transparent. Thin sections are usually required for the examination of materials.

### 2.8. In Vitro Swelling and Degradation Analysis

The swelling test is a key parameter for membrane characterization. First, membrane samples are weighed dry to obtain the dry weight (Wd). The samples are then immersed in PBS or distilled water at 37 °C for a defined period and kept in a shaking incubator. Afterward, excess liquid is removed using a tissue, and the wet weight (Ww) is measured. The swelling ratio (S) is calculated using the following Equation (1).

S = [(Ww − Wd)/Wd] × 100(1)

For the degradation test, scaffold samples are first weighed to obtain the dry weight (Wd), then immersed in PBS or distilled water at 37 °C for a set period. After 24 h of drying at room temperature, the final weight (Wt) is measured. The degradation ratio (D) is calculated using Equation (2).

D = [(Wd − Wt)/Wd] × 100(2)

### 2.9. In Vitro Drug Release Analysis

The produced drug formulation was immersed in 2 mL of phosphate-buffered solution (PBS) at pH 7.4 and gently shaken at 37 °C using a Thermo-Shaker (Yooning, Changzhou, China). At predetermined time intervals, 2 mL of each solution was taken and analyzed by UV-Vis spectroscopy at the wavelength appropriate to the literature for each drug. This amount of solution was replaced with an equal volume of dissolution medium to keep the volume constant. Samples were examined at the wavelengths determined by the UV-Vis spectrometer. Calibration curves and cumulative drug release graphs were drawn for each drug using the obtained results. A UV-Vis spectrophotometer (752N Plus, Istanbul, Türkiye) device was used for cumulative drug release analysis of nanoparticle formulation produced using electrospraying technology.

### 2.10. In Vitro Antibacterial Analysis (Disk Diffusion Test)

The antibacterial properties of the nanoparticle formulation were investigated against *Staphylococcus aureus* (Gram-positive) and *Escherichia coli* (Gram-negative) bacteria using the disk diffusion technique. All the samples were formed into disks of almost the same size. *S. aureus* and *E. coli* bacteria were cultured at 37 °C for 24 h. Then, 0.01 mL of the above-mentioned culture medium was injected into sterilized Petri dishes. A total of 15 mL of Muller-Hinton agar (Merck, Darmstadt, Germany) was given to each infected Petri dish. Disks were placed on the solid agar medium by gently pressing. The treated Petri dishes were incubated at 37 ± 1 °C for 24 h. The inhibitory zones developed on the medium were finally measured. Antibacterial activity experiments were performed in triplicate for each test strain, and average measurements were calculated [24,25].

### 2.11. In Vitro Cytocompatibility Evaluation

The biological characterization of the PCL-Col, PVA/Col/HAp, PCL-Col/PVA/Col/Hap, and PCL-Col/PVA/Col/Hap-Amox composite materials was conducted using the L929 mouse fibroblast cell line. Before the experiment, all specimens underwent UV sterilization for one hour and were subsequently placed into 96-well plates. L929 cells were seeded into each well at a 1 × 10^4^ cells/mL density on the prepared films. The films were then incubated with 5% CO_2_ at 37 °C for 14 days. The growth medium employed consisted of DMEM-low glucose (Dulbecco’s Modified Eagle’s Medium-low glucose), FBS (fetal bovine serum), penicillin/streptomycin, L-glutamine, and Phosphate-buffered saline (PBS) tablets were bought from Amresco (Solon, OH, USA), (99% purity, *v*/*v*), 3-(4,5-dimethyl-2-thiazol)-2,5-diphenyl-2H-tetrazolium bromide (MTT) powder, Trypsin/EDTA solution at 0.25% (*w*/*v*), and dimethylsulfoxide (DMSO) were obtained from Sigma-Aldrich (St. Louis, MO, USA). In vitro cell viability assessment for the L929 mouse fibroblast cells seeded on the films was performed using the MTT assay on the cell culture’s 1st, 3rd, 5th, and 7th days. The growth medium was removed after incubation at 37 °C with 5% CO_2_ for each predetermined day. Subsequently, 90 µL of fresh medium and 10 µL of MTT solution were added to each well, and the incubation was continued for 3 h. After this incubation period, the MTT solution was carefully discarded, and 200 µL of DMSO was added to dissolve the formazan crystals. The composite mats were then incubated for an additional 1 h to ensure complete dissolution. Finally, the media from the wells were taken, and the absorbance values of the solutions were measured via a Dynamic LEDETECT96 microplate reader at 540 nm [26,27].

### 2.12. Statistical Analysis

Statistical differences between groups were analyzed using Two-Way Repeated Measures ANOVA for time-dependent assays (cell viability and drug release) and One-Way ANOVA for single-point measurements. Prior to analysis, the normality of data distribution and homogeneity of variance were verified using Shapiro–Wilk and Levene’s tests, respectively. Post hoc comparisons were performed using the Tukey–Kramer and Dunnett multiple comparison tests. Linear regression analysis was employed to determine the calibration equations and *R*^2^ coefficients for drug release and swelling models. A *p*-value < 0.05 was considered statistically significant. All analyses were performed using GraphPad Prism version 10.

## 3. Result and Discussion

### 3.1. FTIR Analysis

**Figure 1:**
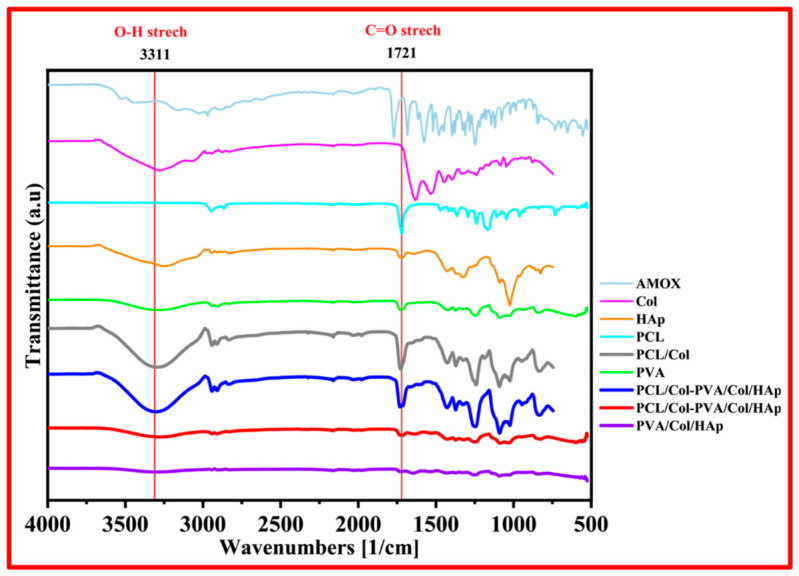
FTIR spectrum of PVA/Col/HAp, PCL/Col-PVA/Col/HAp, and other samples.

The functional groups of the materials used and produced can be determined by FTIR analysis. With this process, the materials used and whether the drug is loaded into the membrane structure can be seen. Firstly, FTIR analysis was performed for the Col and HAp powder materials used. Then, the PVA membrane prepared by dissolving in water and the first layer membrane, PVA/Col/Hap, were analyzed. Afterward, measurements were made for the PCL membrane prepared by dissolving in acetic acid and the second layer membrane, PCL-Collagen. Finally, FTIR analyses were performed with a resolution of 4 cm^−1^ in the scan range from 4000 to 400 cm^−1^ in order to evaluate the possible interactions between the molecules for the powdered amoxicillin drug and PVA-Amox structures produced by dissolving in water. The peak values obtained were compared with the literature. PVA/Col/HAp. Membrane, PCL/Col Membrane, and PVA/Amox particulate electrospray layer, the carbon and elemental structures of the membranes and their constituent materials are shown in Figure 1.

Col characteristic absorption bands are observed around 3284 cm^−1^ [28] for N-H stretching for amide I, C-H stretching at 3073 cm^−1^ [29], a C-H vibration at 2920 cm^−1^, C=O stretching around 1743 and 1630 cm^−1^ [30] for amide I, N-H deformation at 1529 and 1233 cm^−1^ [31], vibration bands at 1159, 970 cm^−1^ [32] associated with C-O-C and 872 cm^−1^ [33] associated with C-O, respectively. FTIR spectra of HAp show typical absorption bands, characteristic O-H stretching ns modes at 3570 cm ^1^ [34], vibration band at 1036 cm^−1^ [35] attributed to ν3 (PO_4_)_3_^−^ groups and ν1 P-O, and bonds of phosphate group at 963 cm^−1^ [36] and hydrogen phosphate group at 875 cm^−1^ [37]. Carbonate groups at 1418 and 1471 cm^−1^ [38] are also present. According to the literature, PCL forms infrared absorption bands related to polymer characterization and modifications. The bands at 2944 cm^−1^ and 2861 cm^−1^ [39] indicate asymmetric and symmetric stretching of CH_2_, respectively. The intense peak at 1725 cm^−1^ is characteristic of the C=O carbonyl group [40]. The main chain of C-O and C-C groups at 1300 cm^−1^ shows symmetric stretching of C-O-C at 1243 cm^−1^ and symmetric stretching of C-O-C groups at 1168 cm^−1^ [41,42]. The spectrum of the HAp-Col composite sample is characterized by absorption bands originating from these two materials. Among the characteristic bands specific to collagen, C=O bands at ∼1635 cm^−1^ [43] for amide I, N-H bands at ∼3305 cm^−1^ for amide A, and C-H bands at ∼3081 cm^−1^ for amide B were observed. For amides I and II, there is N-H deformation at ∼1545 cm^−1^ [41] and phosphate contours as HAp-related bands. The phosphate bands are located between 900 and 1200 cm^−1^ in the IR spectrum. In the HAp spectrum, typical stretching vibration bands of phosphoric groups can be found at 1025 and 1047 cm^−1^. CO_3_ bands are located at ∼1445, 1414, and 876 cm^−1^. In addition, (PO_4_)_3_^−^ bands can be found between 450 and 670 cm^−1^ [44].

The wavelength (cm^−1^) and band information for pure PVA are as follows, according to the literature: the broad band at about 3475 cm^−1^ is the stretching vibration of the hydroxyl group (OH) of PVA. The band corresponding to the CH_2_ asymmetric stretching vibration appears at about 2933 cm^−1^. The peak at 1713 cm^−1^ [45] is attributed to the C=O and the peak at 1658 cm^−1^ [46] to the C=C stretching mode. The absorption peak at 1432 cm^−1^ is the symmetric bending of CH_2_. The band at approximately 1096 cm^−1^ corresponds to the C-O stretching of carbonyl groups in the PVA structure. The C-C stretching vibrations of the moderately absorbed planar zigzag carbon backbone are observed at 844 cm^−1^ [47]. The peak at 651 cm^−1^ is assigned to the sway mode of (OH) groups, while the peak at 921 cm^−1^ is assigned to CH_2_ sway, and the peak at 1332 cm^−1^ corresponds to (CH+OH) bending [48].

Amox main peaks at 3175, 3366, and 3458 cm^−1^ (amide N-H and phenol OH stretching) [49], 3000 cm^−1^ (benzene ring C-H stretching) [50], 2050 cm^−1^ (C≡C and C≡N stretching) [49], 1789 cm^−1^ (β-lactam C=O stretching) [51], 1692 cm^−1^ (amide I, C=O stretching) [49], 1520 cm^−1^ (benzene ring C=C stretching) and 1490 cm^−1^ (N-H bending C-N stretching combination) bands. In the FTIR spectrum for the Amox-containing layer of the fabricated membrane, the characteristic peaks of Amox are also present, with some expansion and decrease in intensity. This shows that Amox has been successfully produced using PVA polymer.

### 3.2. Mechanical Test

**Figure 2:**
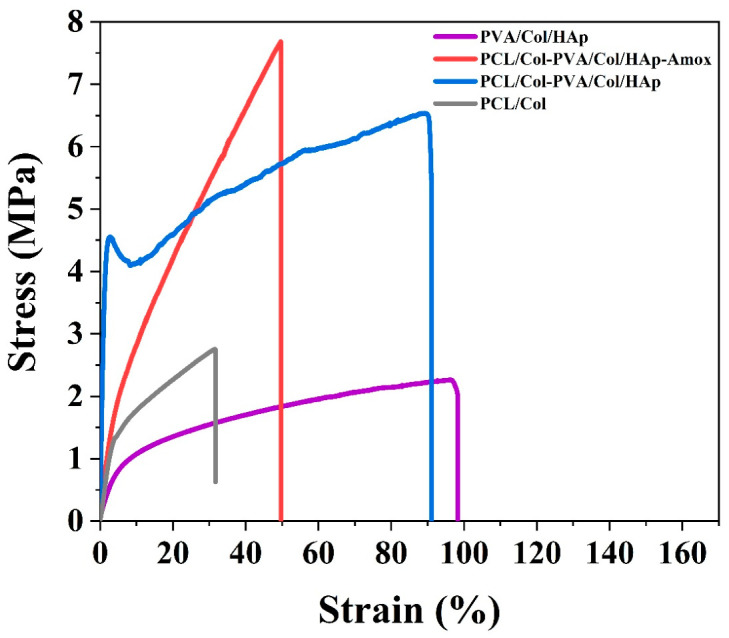
Stress–strain curves of PCL/Col, PVA/Col/Hap, PCL/Col-PVA/Col/Hap, and PCL/Col-PVA/Col/HAp-Amox.

While the maximum stress seen in the PCL graph is 2.05 MPa on average, it is 6.70 MPa on average for PCL-Col. This shows that the addition of collagen increases mechanical strength. In the PCL graph, the elastic region is wider and reaches the breaking point more slowly. This shows that collagen increases the membrane stiffness for PCL-Col (Figure 2).

While PCL is more flexible than PCL-Col membrane, the addition of collagen to the membrane structure decreased the flexibility of the material while increasing its rigidity. The average maximum force was measured as 1.69 N for PCL and 3.60 N for PCL-Col [52], and the values show the strength of the membrane for a certain force. PCL-Col membrane structure is more durable than PCL membrane and is more suitable in terms of mechanical load-carrying capacity. For this reason, it has been observed that it is suitable for use in dental membrane applications. For PCL and PCL-Col., the addition of collagen increased stress. It decreased the strain and elongation rate. This shows that it improves mechanical properties and decreases flexibility. But besides all these, the PCL-Col structure has suitable flexibility for the membrane structure. In total, 20% PVA structure is very brittle and hard, so it disintegrated during the test. The average maximum force was measured as 4.48 N, which shows the strength of the membrane for a given force.

The average maximum stress value was measured as 5.46 MPa. This shows that the membrane has load-carrying capacity. In addition, the average tensile strain is 95.26%. This shows that the membrane structure is flexible.

It is seen that the mechanical and flexibility/elongation properties of the PVA/Col/HAp membrane are suitable for the membrane structure. The average maximum tensile (stress) ratio was measured as 2.16 MPa [49]. The average maximum force was measured as 15.17 N. It is seen that the membrane has the strength for a certain force and has a high load-carrying capacity. The average strain value was measured as 25.24%, and this value shows that the membrane is flexible. It is concluded that the mechanical and flexibility properties of the double-layer membrane are suitable, and as a result of the good bonding of the two layers in the structure, it will meet the dental membrane properties.

The mechanical performance of the developed multilayer membrane is crucial for its clinical application as a barrier in guided bone regeneration (GBR) [53,54,55]. The obtained tensile force of 15.17 N and tensile stress of 2.16 MPa are sufficient to withstand the pressure from overlying soft tissues and the forces applied during surgical handling and suturing. Furthermore, the strain value of 25.24% indicates that the membrane possesses the necessary flexibility to adapt to the complex anatomical contours of alveolar defects without fracturing. When compared to literature, our membrane’s tensile strength is superior to some common electrospun PCL-based scaffolds (typically ranging from 1 to 5 MPa depending on porosity) and falls within the functional range of commercial resorbable membranes [56], which exhibit tensile strengths around 2–7 MPa depending on the hydration state.

### 3.3. SEM-EDS and Optic Analysis

**Figure 3:**
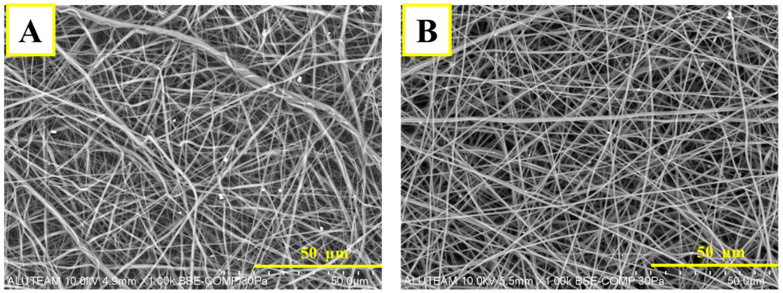
SEM images of (**A**). PCL/Col and (**B**). PVA/Col/HAp layer.

Scanning Electron Microscopy (SEM) analysis for the PVA/Col layer is shown in Figure 3A. As a result of the analysis, it is seen that the structure produced by the electro-spinning method has a nanofiber structure. Since the fiber diameters are equal and homogeneous, it is seen that the parameters are well optimized. The fact that the membrane consists of random fibers shows that it has high porosity. The porous structure facilitates cellular penetration, nutrient, and gas exchange. The bright and white dense parts in the SEM image are hydroxyapatite particles and are also present in the fiber. HAp particles in the SEM image of the membrane composed of PVA/Col/HAp are marked in Figure 3B. The homogeneous fiber structure and the addition of hydroxyapatite particles improved the mechanical and biological performance of the material.

**Figure 4:**
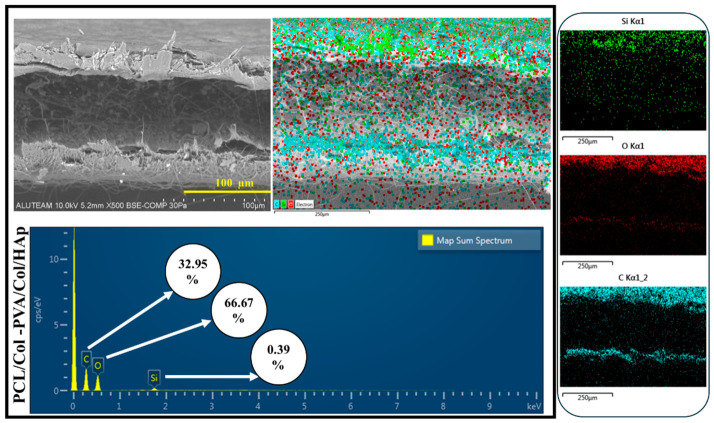
SEM-EDS and Map of PCL/Col-PVA/Col/HAp.

EDS for the P PCL/Col-PVA/Col/HAp layer is shown in Figure 4. C (Carbon) is shown in blue in the image. It is the main component of PVA and Collagen and is present in the majority of the fiber structure. O (Oxygen), shown in red in the image. It is present in the structure of hydrophilic PVA and Col. It is also present in the hydroxyapatite structure, indicating that the membrane has a uniform composite structure [51]. The distribution of oxygen shows that the membrane structure is hydrophilic. Therefore, it will facilitate the interaction of biological fluids and cells. The orange color in the image refers to Ca (Calcium). This indicates the presence of hydroxyapatite. When found with phosphorus, it forms bone-like structures. P (Phosphorus), shown in purple in the image. Phosphorus is one of the main components of hydroxyapatite. It is seen together with calcium. The fact that Ca and P structures are seen in similar regions indicates that hydroxyapatite is well integrated into the structure [13,23,57]. It can also be said that hydroxyapatite is homogeneously present in the membrane and will increase biomimetic properties while providing mechanical strength. It also allows osteoblasts (bone cells) to attach and grow on the membrane. In the SEM image of the membrane layer composed of PCL-Col (Figure 3), it is seen that the structure consists of nanofibers. According to the EDS analysis shown in Figure 4, it is seen that C (Carbon), the main component of the PCL structure, is homogeneously distributed. Collagen is a protein containing both C (Carbon) and oxygen. The presence of oxygen in the structure indicates that collagen is present in the fibers, and a good mixture is made.

The SEM Image taken from the top surface of the double-layer membrane structure (PCL/Col-PVA/Col/HAp) is shown in Figure 4. The top layer consists of a nanofiber structure composed of PVA/Collagen/Hydroxyapatite structure. It is seen from the image that the surface has a fiber structure. Figure 4 shows the side definitive SEM images for the double-layer structure. EDS analysis for this structure is shown in Figure 4. According to this analysis, the top layer is the PVA/Col/HAp layer [51,58,59]. The bottom layer shows the PCL/Col structure. The amount of Oxygen (O) is lower in the lower layer structure and is due to Collagen. It is also seen this way because PCL is hydrophobic. Carbon (C) is present in both layers. In the PVA/Collagen/Hydroxyapatite layer, C is concentrated because hydroxyapatite is present. Since PCL is a carbon-based polymer, it is also present in the bottom layer. It can be seen that the two membrane layers come together harmoniously, and there is a bonding between the layers. It can also be said that good bonding of the layers reduces the risk of delamination and provides long-term stability [60].

**Figure 5:**
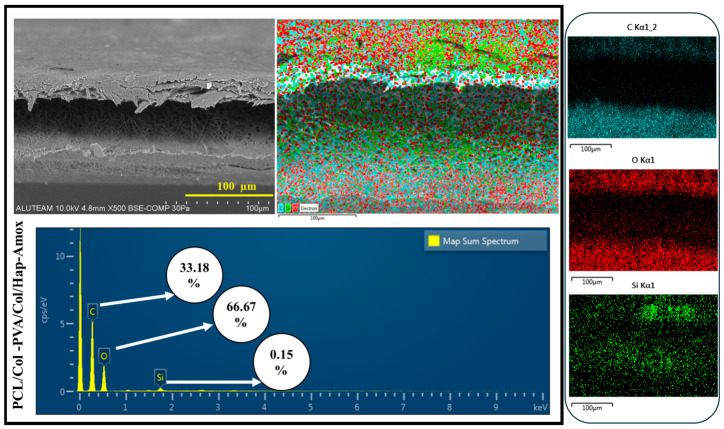
SEM-EDS and Map of PCL/Col-PVA/Col/Hap-Amox.

The electrospray process applied to the double-layer structure (PCL/Col-PVA/Col/HAp-Amox) is given in the image in Figure 5. EDS analysis for the electrosprayed double-layer membrane structure is shown in Figure 5. As a result of the analysis, the presence of Carbon and Oxygen density in the top layer indicates that the electrospray process was performed.

**Figure 6:**
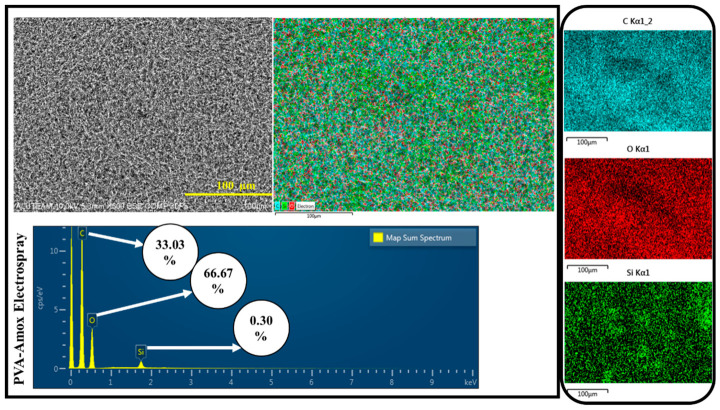
SEM-EDS and Map of PVA-Amox electrospray.

SEM analysis for the PVA/Amox Electrospray Layer is shown in Figure 6, and there are particulate structures on the surface as a result of the electrospray process. According to the EDS analysis for the electrospray layer, there is a high amount of Carbon (C) originating from the PVA polymer. Amoxicillin also contributes to these signals since it has an organic structure. Oxygen (O) is due to PVA and Amoxicillin [61,62,63].

**Figure 7:**
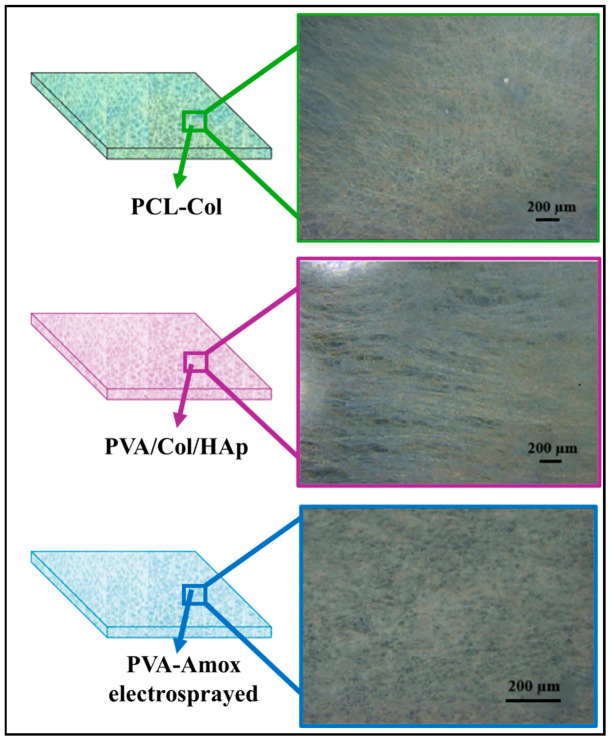
Membrane Layers and Optical Microscope Images.

During the membrane production with the electro-spinning device, the structure analysis was first examined with an optical microscope. Membrane layers and optical microscope images are shown in Figure 7. The optical microscope image for the membrane layer composed of PCL-Collagen is given in Figure 7. It is seen that the membrane structure consists of fibers.

The optical microscope image of the micro-sized particles produced from PVA-Amox by the electrospraying method is shown in Figure 7. With this method, it was observed that the particles containing antibiotic drugs were properly distributed over the entire surface.

### 3.4. In Vitro Swelling-Degradation

The swelling and degradation behaviors of the membranes were investigated with 3 mg samples for PCL/Collagen (Col.) and double-layer membrane (PCL/Col-PVA/Col/HAp). Experimental data were obtained using three samples for each hour interval. Swelling behavior was investigated for 1, 2, 3, 6, 9, 9, 12, 24, 36, 48 h, and the swelling value was calculated according to Equation (1). The degradation behavior was investigated for 1, 6, 9, 12, 24, 36, and 48 h; the degradation value was calculated according to Equation (2), and graphs were created.

**Figure 8:**
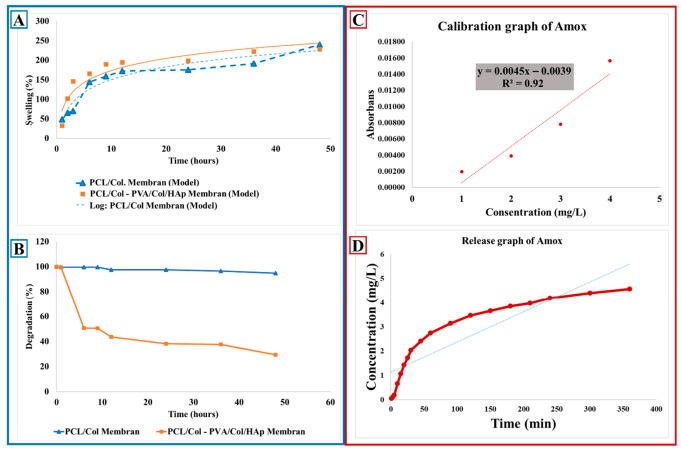
(**A**) swelling behavior graph with experimental values and mathematical models of membranes produced by the hybrid production technique, (**B**) degradation behavior for PCL-COL. membrane and double layer (PCL/COL-PVA/COL/HAp) membrane, (**C**) amoxicillin calibration graph, (**D**) drug release graph of double-layer membrane (PCL/Col-PVA/Col/HAp-PVA/Amox.) containing Amoxicillin charged particles.

When the swelling behavior was examined, in the PCL/Col-PVA/Col/HAp double-layer structure, the layer containing PVA degraded rapidly, and a sudden swelling behavior occurred in the membranes due to collagen. Afterward, the swelling behavior increased rapidly and reached swelling equilibrium within 6 h (Figure 8A). PCL/Col Membrane structure reached a swelling rate of approximately 150% by the 6th hour, and a rapid absorption was realized. This can be attributed to the ability of collagen content to rapidly absorb water. After the 6th hour until the 48th hour, swelling behavior reached a constant level and stabilized, and membrane stabilization was achieved. With this behavior, it is seen that the water holding capacity of the membrane structure increases over time, and then stabilization is observed. This may be a positive sign for long-term biological stability [51,64].

According to the swelling behavior of the double-layer membrane consisting of PCL/Col-PVA/Col/HAp layers, it was observed that there was a rapid increase in the swelling rate of the membrane up to approximately 100% until 120 min. It was observed that the swelling rate of the membrane increased rapidly from 120 min to 6 h, and then the increase slowed down to 48 h.

Degradation behaviors for PCL/Col. and double-layer (PCL/Col-PVA/Col/HAp) membranes are examined in Figure 8B. While examining the degradation behaviors, a rapid weight loss was observed in the PVA-containing layer in the bilayer structure (PCL/Col-PVA/Col/HAp). This is due to the low resistance of PVA to aqueous media and its tendency to dissolve rapidly. The rapid degradation of the PVA-containing layer is due to the hydrophilic nature of PVA. The early degradation of this layer caused the membrane to initially absorb too much liquid, and the degradation started rapidly. A crosslinker can be added to prevent the degradation of PVA. It was observed that the weight loss of the double-layer membrane was approximately 4% within 48 h, and the degradation behavior stabilized with time. This is related to the more durable nature of the PCL content of the membrane. However, the weight loss observed in the PCL/Col. layer is due to the fact that the dissolution rates of collagen are different from those of PCL. This may be due to the fact that collagen is more sensitive to water and has a high liquid absorption due to its porous structure. In addition, the loss in the weight of the double-layer PCL/Col-PVA/Col/HAp membrane may be due to the fact that collagen accelerates degradation by increasing its sensitivity to degradation and water retention capacity [49,61]. It was observed that the thickness differences in the layers during the electrospinning method may also be effective in this behavior. Since the degradation time of PCL polymer is long, it can be said that the degradation behavior is fixed after 60 min. The stabilization of the degradation rate in the double-layer membrane after 60 min is due to the long biodegradation time of PCL. This is important for the long-term biological stability of the membrane. Although PCL polymer is biodegradable, it can remain stable in biological environments because it has long-term degradation resistance.

Collagen is more rapidly degradable than PCL and has been observed to accelerate degradation. However, this property may provide an advantage for the rapid initialization of the membrane in biological applications. In the PCL/Collagen combination, the water sensitivity of collagen and the porous structure caused a rapid initial degradation. The degradation behavior of the PCL/Col-PVA/Col/HAp membrane structure first shows a rapid increase and then slows down, which may also be due to the different degradation rates of the membrane surface and layers. Although the membrane degradation level is fast in the first hours, it slows down and stabilizes over time [65,66,67].

It has been stated that the different degradation rates seen between the membrane surface and layers at first may be due to thickness differences in the design of the membrane. However, the slowing and stabilization of degradation over time prove that the membrane exhibits biodegradability in a controlled manner. Different layer properties of bilayer membranes may be advantageous to optimize the overall degradation behavior. In addition, in dental applications, it provides advantages such as creating time to allow bone regeneration or allowing the regeneration of the gingival structure.

### 3.5. In Vitro Release Analysis

UV-Vis. With the results obtained in the spectrophotometer, a drug release graph was generated in Figure 8C. According to the drug release graph of the double-layer membrane containing Amox particles, the drug concentration increased rapidly within the first 50 min. This indicates that drug release occurred rapidly in the beginning. After 50 min, the concentration increase in Amoxicillin slowed down. This indicates that the drug release rate started to approach equilibrium after 50 min. At 350 min, the drug concentration reached approximately 4.5 mg/L. At this point, the graph was horizontal, indicating that the release had reached a steady level. As a result, it shows that initially the drug is released rapidly, then the release rate slows down, reaches a constant rate, and then reaches equilibrium over time.

The drug was sprayed with PVA/Amoxicillin solution by the electrospray method on a double-layer membrane consisting of PCL/Col-PVA/Col/HAp. Amoxicillin drug release analysis was performed for the dental membrane (PCL/Col-PVA/Col/HAp-PVA/Amox.) containing particles loaded with amoxicillin drug. The drug release test for the produced membrane was carried out with a shaking incubation device (Thermo-Shaker) at 37 °C. In the analysis, 2 mL of PBS (pH = 7.4) was used for each ependorf [49,68].

In order to compare the values obtained during drug release from the membrane, a calibration curve was drawn by measuring different dilution ratios of amoxicillin with water-amoxicillin solution prepared with Amoxicillin (Figure 8D). For the absorbance value obtained, the graph in Figure 8C shows that as the concentration of amoxicillin increases, the absorbance value of the solution increases. It is seen that there is a linear relationship between absorbance and concentration. The equation on the graph is y = 1.5827x + 0.06 and R^2^ = 0.9957. Since the R^2^ value is close to one, it is seen that the calibration curve represents a strong linear relationship [61,69].

For drug release analysis, firstly, one PBS tablet (Phosphate-Buffered Saline) was placed in 100 mL of pure water and mixed in a magnetic stirrer. The fully dissolved PBS solution with pH = 7.4 was transferred to ependorfs, 2 mL each. Then, three samples of 20 mg each were prepared from an electrosprayed double-layer membrane (PCL/Col-PVA/Col/HAp-PVA/Amox.). The samples were left completely immersed in PBS. Each membrane in the Ependorf was set to 37 °C for 1, 3, 5, 10, 15, 15, 20, 25, 25, 30, 45, 60, 60, 90, 90, 120, 120, 150, 180, 180, 210, 240, 300, and 360 min and placed in a shaker incubator. At each minute interval, the PBS solution was removed from the ependorf and 2 mL of PBS solution was added again, and the analysis was continued. The times for the separated samples were labeled on the ependorf. Measurements for the PBS in each ependorf were performed on UV-Vis. Spectroscopy device with a wavelength of 275 nm.

### 3.6. Antibacterial Analysis

In the disk diffusion test, as the drug diffuses, a concentration gradient is formed. The presence of the drug creates zones of inhibition that inhibit the growth of the microorganism. Zones of inhibition diameters are categorized into sensitivity categories according to clinical breakpoints, which are updated regularly. Several factors influence zone diameters, which may be related to the drug (disk content, diffusion rate, activity against the tested isolate), agar (depth, composition), incubation conditions (temperature, time, atmosphere), or microorganism (growth rate, inoculum density).

An antibacterial test was performed for PCL/Col-PVA/Col/HAP-PVA/Amox. membrane produced in a double layer and electrosprayed with the drug amoxicillin.

**Figure 9:**
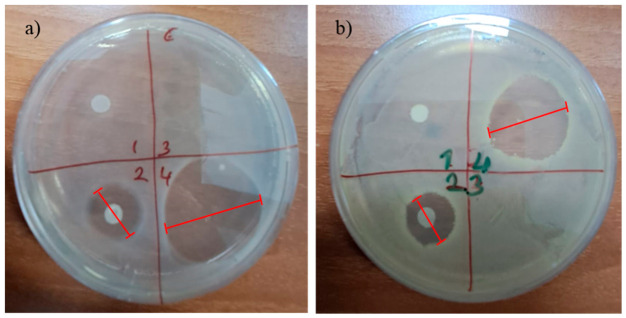
Antibacterial Test (**a**) *E. coli* (Gr−), (**b**) *S. aureus* (Gr+).

In order to evaluate the antibacterial properties of the double-layer membrane structure electrosprayed with antibiotic and the membrane structure without electrospraying, PVA solution used for electrospraying and PVA-Amoxicillin solvent cast films, the effect on the viability of *E. coli* (Gr−) and *S. aureus* (Gr+) bacteria was examined in vitro using the disk diffusion method. Double-layer membrane and solvent cast films without antibiotic loading were used as negative controls, and the zone diameters of the drug-loaded structures formed relative to the negative control were monitored by measuring the zone diameters (Figure 9). After 24 h of incubation, the diameters of the zones of inhibition formed around the membrane and solvent casting applied as disks were measured.

The in vitro antibacterial capacity of films prepared with a double-layer membrane and electrospray solution against *S. aureus* (Gram-positive bacteria) and *E. coli* (Gram-negative bacteria) was evaluated using the disk diffusion method. The electrosprayed membrane (PCL/Col-PVA/Col/HAP-PVA/Amox.) and the solvent film of the electrospray solution formed zones of inhibition and showed activity against the bacteria used in the test. As a result of the test, non-electrosprayed double-layer membrane (PCL/Col-PVA/Col/HAP) and electrosprayed PVA solution film without amoxicillin were used as negative controls and did not show antibacterial activity against *S. aureus* and *E. coli* bacteria. The electrosprayed membrane (PCL/Col-PVA/Col/HAP-PVA/Amox.) and the solvent film of the electrospray solution formed an inhibition zone and showed antibacterial activity against the bacteria used in the test [49,50,70].

### 3.7. Evaluation of Cytocompatibility

**Figure 10:**
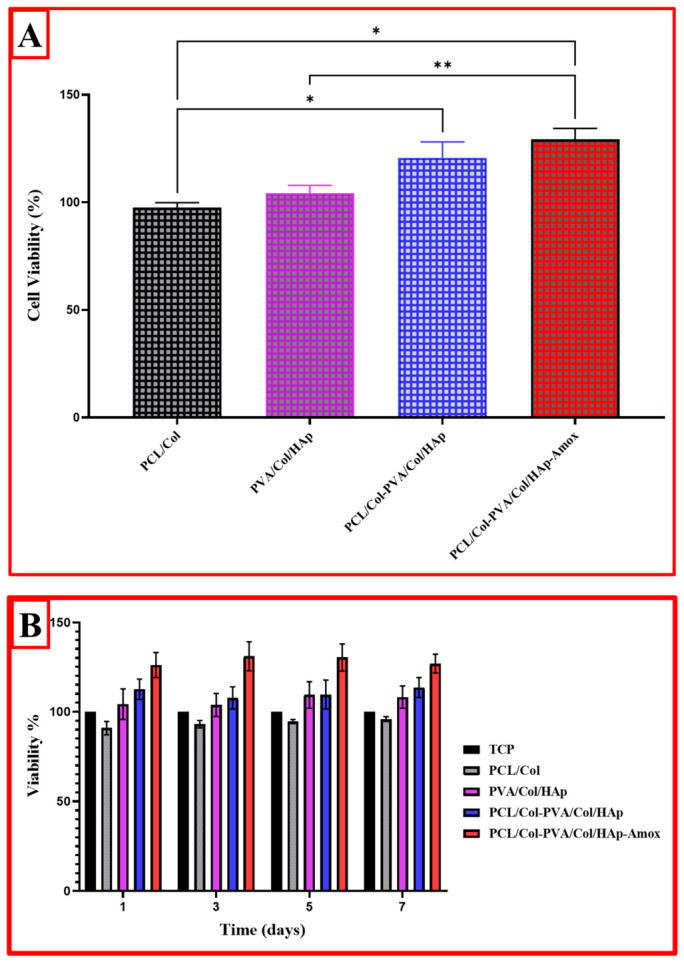
(**A**). Cell viability results are given as % of the negative control, mean ± SD values of n = 3 independent experiments. * *p* < 0.001, and ** *p* < 0.0001 vs. negative control group. Data were analyzed using Two-Way Repeated Measures ANOVA. (**B**). Cell viability of L929 cells exposed to different concentrations (days 1, 3, 5, and 7) of PCL and PVA-based composites.

In this study, the cellular biocompatibility and cytotoxicity profiles of PCL and PVA-based biomaterials were investigated in detail. The cell viability results allow a comparative evaluation of the effects of various combinations of PCL and PVA on cell proliferation. When the cell viability results in the first graph are analyzed, it is seen that the PVA/Col/HAp group made a positive contribution of 5–10% to cell viability when the PCL/Col group was taken as a reference control. This may be attributed to the cellular adhesion-enhancing effect of hydroxyapatite (HAp). The PCL/Col-PVA/PVA/Col/HAp group showed approximately 15–20% higher cell viability compared to the PCL/Col group (*p* < 0.05), indicating that PCL and PVA better support cellular interaction when used in combination (Figure 10A). The most remarkable result was that the PCL/Col-PVA/Col/HAp-Amox group achieved approximately 25–30% higher cell viability (*p* < 0.01). This suggests the role of Amoxicillin as both an antibacterial and a cell proliferation-promoting factor. Especially when compared to the PCL/Col group, this increase was statistically significant (*p* < 0.01, *p* < 0.05), indicating that the addition of the antibacterial agent did not inhibit cell growth but rather promoted it [49,52,58].

When time-based cell viability results were analyzed, it was observed that cell viability rates of all groups ranged between 90 and 110% on day 1. While PCL/Col and TCP groups showed approximately 90–95% cell viability, this rate increased to 105–110% in PVA/Col/HAp and PCL/Col-PVA/Col/HAp groups (Figure 10B). It is observed that cell proliferation increased on the fifth day in all groups. The PCL/Col-PVA/Col/HAp-Amox group showed the highest biocompatibility, reaching approximately 130–135% viability on day 5. While the PCL/Col group remained at 100% in this process, 115–120% and 120–125% viability rates were observed in the PVA/Col/HAp and PCL/Col-PVA/Col/HAp groups, respectively.

When the data were analyzed, the PCL/Col-PVA/Col/HAp-Amox group showed the highest proliferation with a cell viability value close to 140%, indicating that the material creates a supportive microenvironment in the cellular environment. The other groups reached PCL/Col (100%), PVA/Col/HAp (110–115%), and PCL/Col-PVA/Col/HAp (125–130%), respectively. These results show that PCL and PVA-based materials are biocompatible even when used alone, but the addition of additives such as hydroxyapatite and Amoxicillin significantly increased cell proliferation. In particular, the PCL/Col-PVA/Col/HAp-Amox group had the highest cell viability, suggesting that this combination provides the most favorable environment for cellular growth and adhesion. This suggests that antibacterial agent-doped biomaterials may not only provide protection against pathogens but also have a cell growth-promoting effect [64,71,72].

In conclusion, the data obtained show that PCL and PVA-based composites are promising for biomedical applications. Especially the PCL/Col-PVA/Col/HAp-Amox group has high potential in the fields of tissue engineering and regenerative medicine, with both biocompatible and antibacterial properties, and needs to be further tested for in vivo applications.

## 4. Conclusions

In this study, a novel multilayered dental membrane was successfully fabricated using electrospinning and electrospraying techniques, integrating polycaprolactone (PCL), polyvinyl alcohol (PVA), collagen, hydroxyapatite, and amoxicillin. Quantitative evaluations confirmed the membrane’s favorable mechanical integrity, with a maximum tensile force of 15.17 N and strain of 25.24%, indicating sufficient flexibility and strength for intraoral applications. The dual-layer structure exhibited controlled swelling behavior, reaching saturation within 6 h, and a degradation profile stabilizing after an initial 4% weight loss within 48 h. Drug release kinetics demonstrated a rapid initial release phase, reaching a plateau at ~4.5 mg/L amoxicillin after 350 min, suggesting effective antimicrobial dosing. The membrane exhibited significant antibacterial efficacy against both *S. aureus* and *E. coli*, supported by clearly defined inhibition zones in disk diffusion tests. Cytocompatibility assays using L929 fibroblasts revealed enhanced proliferation, particularly in the amoxicillin-loaded group, which achieved ~140% cell viability, indicating the membrane’s potential to support tissue regeneration. Collectively, these quantitative findings validate the developed membrane as a multifunctional, biocompatible, and resorbable scaffold, with promising applications in guided tissue and bone regeneration within dental and maxillofacial procedures.

## Data Availability

The original contributions presented in this study are included in the article. Further inquiries can be directed to the corresponding author.
